# Patient and provider experience and perspectives of a risk-based approach to multidisciplinary chronic kidney disease care: a mixed methods study

**DOI:** 10.1186/s12882-019-1269-2

**Published:** 2019-03-29

**Authors:** Michelle D. Smekal, Helen Tam-Tham, Juli Finlay, Maoliosa Donald, Chandra Thomas, Robert G. Weaver, Robert R. Quinn, Kin Tam, Braden J. Manns, Marcello Tonelli, Aminu Bello, Navdeep Tangri, Brenda R. Hemmelgarn

**Affiliations:** 1 Department of Medicine, Cumming School of Medicine, University of Calgary, Calgary, Alberta Canada; 2 Department of Community Health Sciences, Cumming School of Medicine, University of Calgary, Calgary, AB Canada; 3 Southern Alberta Renal Program, Alberta Health Services, Calgary, Alberta Canada; 4 Interdisciplinary Chronic Disease Collaboration, Calgary, Alberta Canada; 5 Department of Medicine, University of Alberta, Edmonton, Alberta Canada; 6 Department of Community Health Sciences, University of Manitoba, Winnipeg, Canada; 7 Department of Internal Medicine, University of Manitoba, Winnipeg, Canada; 8 Chronic Disease Innovation Centre, Seven Oaks General Hospital, Winnipeg, Manitoba Canada

**Keywords:** Chronic kidney disease, Kidney failure, Kidney failure risk, Non-dialysis care, Mixed methods research

## Abstract

**Background:**

The Kidney Failure Risk Equation (KFRE) predicts risk of progression to kidney failure and is used to guide clinical decisions for patients with chronic kidney disease (CKD).

**Methods:**

The KFRE was implemented to guide access to multidisciplinary care for CKD patients in Alberta, Canada, based on their 2-year risk of kidney failure. We used a mixed methods approach to investigate patients’ and providers’ perspectives and experiences 1 year following KFRE implementation. We conducted post-implementation interviews with multidisciplinary clinic providers and with low-risk patients who transitioned from multidisciplinary to general nephrology care. We also administered pre- and post-implementation patient care experience surveys, targeting both low-risk patients discharged to general nephrology and high-risk patients who remained in the multidisciplinary clinic, and provider job satisfaction surveys.

**Results:**

Twenty-seven interviews were conducted (9 patients, 1 family member, 17 providers). Five categories were identified among patients and providers: targeted care; access to resources outside the multidisciplinary clinics; self-efficacy; patient reassurance and reduced stress; and transition process for low-risk patients Two additional categories were identified among providers only: anticipated concerns and job satisfaction. Patients and providers reported that the risk-based approach allowed the clinic to target care to those most likely to experience kidney failure and most likely to benefit from multidisciplinary care. While some participants indicated the risk-based model enhanced the sustainability of the clinics, others expressed concern that care for low-risk patients discharged from multidisciplinary care, or those now considered ineligible, may be inadequate.

Overall, 413 patients completed the care experience survey and 73 providers completed the workplace satisfaction survey. The majority of patients were satisfied with their care in both periods with no overall differences. When considering the responses “Always” and “Often” together versus not, there were statistically significant improvements in domains of access to care, caring staff, and safety of care. There were no differences in healthcare providers’ job satisfaction following KFRE implementation.

**Conclusions:**

Patients and healthcare providers reported that the risk-based approach improved the focus of the multidisciplinary CKD clinics by targeting patients at highest risk, with survey results suggesting no difference in patient care experience or healthcare provider job satisfaction.

**Electronic supplementary material:**

The online version of this article (10.1186/s12882-019-1269-2) contains supplementary material, which is available to authorized users.

## Background

Chronic kidney disease (CKD) affects approximately 12% of adults in Canada with significant morbidity and mortality [1]. CKD patients often receive suboptimal care [2], partly because identifying patients at highest risk of progression is challenging [3]. Although models to predict risk of progression have existed for many years [4, 5], the recently validated 4-variable kidney failure risk equation (KFRE) [6, 7] allows for a more accurate assessment of risk and has been implemented in a variety of clinical settings [8–11]. However, evaluation of the KFRE’s use as a tool to guide triage and care delivery for CKD patients has been limited.

In the past 20 years, many centres have established multidisciplinary clinics to care for patients with advanced CKD in a team-based setting, however the evidence to support the effectiveness of this resource-intensive care has been limited [12]. The KFRE provides the opportunity to identify patients at higher risk of progression, and tailor their care accordingly. We previously reported a qualitative description of patients’, family members’, and providers’ perceptions of the KFRE [13], which informed our implementation strategy. This follow-up study provides a description of patients’ and providers’ experiences and perceptions 1 year following KFRE implementation, as well as assessments of patient care experience and provider job satisfaction before and 1 year after implementation. Our goals were to better understand the use of the KFRE in clinical decision-making, evaluate patient experience and provider job satisfaction pre/post implementation, and assess the risk-stratification implementation process from a quality improvement perspective.

## Methods

**Fig. 1:**
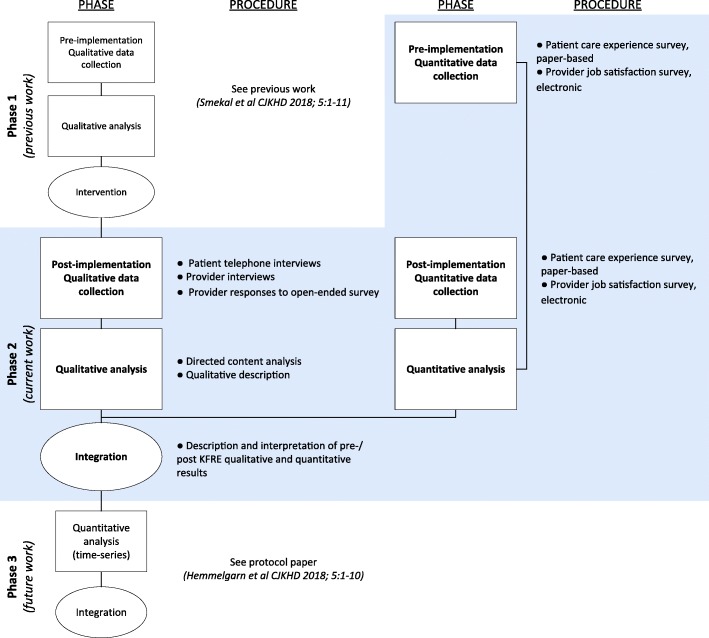
Kidney Failure Risk Equation (KFRE) Implementation multiphase study design. Blue box denotes focus of this paper

This study is one phase of our multiphase study evaluating use of the KFRE to guide CKD care in Alberta, Canada [14]. We report a concurrent mixed methods design [15] to describe and compare patients’ and providers’ experience 1 year following KFRE implementation, as well as report on patient care experience and provider job satisfaction before and after implementation (Fig. 1). Ethics approval was granted by the University of Calgary Conjoint Health Research Ethics Board.

### Study setting

The CKD multidisciplinary clinic provides team-based care to patients with advanced CKD. The primary goals of the clinic are to slow disease progression, manage symptoms and comorbidities, and prepare patients for renal replacement therapy. Beginning in 2017, the KFRE was used as a tool to guide access to multidisciplinary CKD care for all patients residing in the southern regions of Alberta, Canada. Patients at higher risk of kidney failure (2-year KFRE ≥10% or eGFR ≤15 ml/min/1.73m^2^) were recommended to receive multidisciplinary care, including a nurse case manager, dietitian, pharmacist, and social worker, while lower-risk patients (2-year KFRE < 10%) were recommended to receive care from general nephrology (remaining under the care of their primary nephrologist with referral-based access to allied health professionals, but without a nurse case manager).

Our pre-implementation findings [13] revealed both patient and provider concerns relating to care experience and quality for lower risk patients who would be risk-stratified out of the multidisciplinary clinic, or considered ineligible, following KFRE implementation. Consequently, our study focuses on the perspectives and experiences of low-risk multidisciplinary clinic patients who were discharged from multidisciplinary care following KFRE implementation and explores satisfaction of these low-risk patients as well as satisfaction of higher-risk patients who remain in, or were referred to, the multidisciplinary clinic during the study timeframe.

We conducted the patient care experience and provider job satisfaction surveys pre- and post-implementation to broadly assess potential changes to experience or satisfaction following this change to clinic operations.

### Post-implementation - qualitative component

#### Patient and provider selection and recruitment

English-speaking, adult (age ≥ 18 years) non-dialysis CKD patients who were deemed low risk and discharged from multidisciplinary CKD care to general nephrology care following KFRE implementation in the Southern Alberta Renal Program were eligible to participate in a semi-structured telephone interview. Clinic staff screened patients in Calgary, Alberta, for eligibility and obtained consent for contact by a research coordinator. All multidisciplinary CKD healthcare providers (nephrologists, nurses, and allied health professionals) in Calgary, Alberta, were invited to participate by e-mail.

#### Data collection

Two investigators (M.D.S and H.T.) with qualitative research experience conducted all interviews. We used open-ended questions to explore benefits and challenges to KFRE implementation as well as patient and provider experiences transitioning from the CKD multidisciplinary clinic to general nephrology practice (Additional files 1 and 2). Patient and provider interviews were approximately 20 min. All interviews were audio-recorded and transcribed verbatim. Data collection was stopped once we reached data saturation, when no new additional concepts informing the research objectives were identified [16].

Four open-ended questions related to the benefits and challenges of KFRE implementation were completed by healthcare providers as part of the on-line survey, described below, and incorporated in the qualitative analysis (Additional file 3).

#### Qualitative data analysis

We summarized patients’, family members’ and providers’ perspectives from interviews and open-ended responses from provider surveys. Text data was imported into NVivo Version 12 software to facilitate data analysis (QSR International Ltd., Doncaster, Australia). Based on findings from the pre-implementation qualitative study [13], we applied a directed content analysis [17] approach to focus the interview guides, identify relationships between the pre- and post-implementation code definitions and to qualitatively describe [18] participant perspectives; we did not apply a theory to guide analysis. We also asked open-ended questions to identify new content categories and extend our conceptual framework relating to the benefits and challenges of a risk-based approach to multidisciplinary CKD care [17].

Three investigators (M.D.S, H. T, and J.F) independently reviewed the transcripts and developed a coding scheme guided by relevant findings from the pre-implementation qualitative study. The investigators went through several iterations of transcript review and coding scheme refinement to ensure the full range of data was considered (investigator triangulation [19, 20]) and until consensus was reached.

### Pre and post-implementation - quantitative component

#### Patient and provider selection and data collection

All patients attending the CKD multidisciplinary clinics in Calgary as well as low-risk patients discharged from multidisciplinary care to general nephrology care following implementation of the KFRE were eligible to complete an anonymous, paper-based care experience survey (the Care Experience Feedback Improvement Tool [CEFIT]) [21] . Clinic staff provided the survey to eligible patients for completion during clinic visits. The surveys were distributed from November 2016 through January 2017 (pre-implementation) and January 2018 through March 2018 (post-implementation).

All multidisciplinary CKD healthcare providers (nephrologists, nurses, and allied health professionals) from the multidisciplinary clinics in Calgary were invited by email to participate in an online anonymous job satisfaction survey (the Andrews and Withey Job Satisfaction questionnaire [22]). Survey responses were collected online from November 2016 through January 2017 (pre-implementation) and in April 2018 (post-implementation) (OutSideSoft Solutions, Inc., Quebec, Canada).

The patient and provider surveys were collected anonymously and therefore it was not possible to link pre- and post-implementation survey responses.

#### Quantitative data analysis

Nonparametric statistics were used for the Likert-type survey data [23]; pre- and post-implementation differences in survey responses were compared using the Wilcoxon Mann-Whitney rank sum test for unmatched data [24, 25]. Analyses were performed using Stata version 14 (StataCorp, College Station, TX).

## Results

### Qualitative component

#### Patient and provider characteristics

Of the 23 eligible patients approached by clinic nurses to obtain consent for contact by the research team, 13 consented to be contacted and 10 refused. Of the 13 patients contacted by the research team, nine provided consent and participated in a telephone interview; one family member self-referred and participated in a telephone interview (Table 1). Participants were all low-risk (2-year KFRE < 10%) patients discharged from the multidisciplinary clinic following KFRE implementation and followed by their nephrologist only. The majority of participants were ≥ 65 years of age and had previously spent > 5 years as patients in the multidisciplinary clinic. Males and females participated equally and the majority reported their health status as ‘good’ or ‘very good’.

**Table 1:** Demographic characteristics of patient participants (pre- and post-implementation surveys and post-implementation interviews)

Characteristic	Pre-implementation	Post-implementation		
	Survey n (%)	Survey n (%)	P^†^	Interviews n (%)
Overall participation	*n* = 176	*n* = 237		*n* = 10
Participant Type				
Patient	176 (100)	237 (100)	n/a	9 (90)
Family Member	0 (0)	0 (0)		1 (10)
Gender				
Male	106 (60)	136 (57)	0.49	5 (50)
Female	67 (38)	99 (41)		5 (50)
Other	3 (2)	2 (1)		
Age				
< 50	18 (10)	41 (17)	0.03	0 (0)
50–64	50 (28)	44 (19)		1 (10)
65 to 74	39 (22)	63 (27)		3 (30)
≥ 75	69 (39)	89 (38)		6 (60)
Years at CKD Clinic^a^				
< 1	22 (13)	50 (21)	0.02	0 (0)
1 to 5	99 (57)	136 (58)		1 (11)
> 5	54 (31)	47 (20)		8 (89)
Did not answer	1 (1)	4 (2)		0 (0)
Marital Status				
Single	16 (9)	29 (12)	0.40	0 (0)
Married	116 (66)	135 (57)		7 (70)
Widowed	23 (13)	32 (14)		2 (20)
Divorced	11 (6)	25 (11)		1 (10)
Separated	5 (3)	9 (4)		0 (0)
Common Law	4 (2)	7 (3)		0 (0)
Did not answer	1 (1)	0 (0)		0 (0)
Employment Status				
Full-time	20 (11)	44 (19)	0.23	1 (10)
Part-time	14 (8)	15 (6)		0 (0)
Retired	112 (64)	145 (61)		8 (80)
Not employed	19 (11)	21 (9)		1 (10)
Other	9 (5)	12 (5)		0 (0)
Did not answer	2 (1)	0 (0)		0 (0)
Level of Education				
< grade 12	36 (21)	46 (19)	0.67	0 (0)
High School Diploma	49 (28)	54 (23)		2 (20)
Post-secondary	70 (40)	111 (47)		6 (60)
Graduate School	19 (11)	24 (10)		2 (20)
Did not answer	2 (1)	2 (1)		0 (0)
Self-reported health Status^a^				
Poor	19 (11)	25 (11)	0.37	0 (0)
Fair	61 (35)	80 (35)		3 (33)
Good	76 (43)	87 (35)		2 (22)
Very Good	17 (10)	35 (15)		4 (44)
Excellent	2 (1)	5 (2)		0 (0)
Did not answer	1 (1)	5 (2)		0 (0)

^a^Family member not included

†Chi square test; Pre- and post-implementation comparison

Of the 75 healthcare providers eligible for an interview, 17 responded to the email invitation and participated in an interview (Table 2). The majority of participants were female and had worked in their profession for > 10 years with > 5 years experience working at the multidisciplinary clinic. Of the 33 healthcare providers responding to the online survey, 27 completed the open-ended questions included in the qualitative analysis.

**Table 2:** Demographic characteristics of healthcare provider participants (pre- and post-implementation surveys and post-implementation interviews)

Characteristic	Pre-implementation	Post-implementation		
	Survey n (%)	Survey n (%)	P^†^	Interview n (%)
Overall participation	*n* = 40	*n* = 33		*n* = 17
Gender				
Male	11 (28)	9 (27)	0.12	5 (29)
Female	29 (73)	19 (58)		12 (70)
Other	0 (0)	3 (15)		0 (0)
Did not answer	0 (0)	2 (6)		
Provider Role				
Nephrologist	15 (38)	15 (45)	0.41	6 (35)
Nurse	12 (30)	10 (30)		8 (47)
Allied Health/Other	13 (32)	8 (24)		3 (17)
Years in Profession				
< 5	7 (18)	2 (6)	0.10	2 (11)
5–10	7 (18)	12 (36)		3 (17)
> 10	26 (65)	19 (58)		12 (70)
Years at CKD Clinic				
< 1	1 (3)	1 (3)	0.97	1 (5)
1–5	13 (33)	10 (30)		4 (23)
> 5	26 (65)	22 (67)		12 (70)

^†^Chi square test; Pre- and post-implementation comparison

#### Content categories

Based on the patient and provider interviews we categorized participant perspectives into seven content categories: targeted care; access to resources outside the multidisciplinary clinics; self-efficacy; patient reassurance and reduced stress; transition process for low-risk patients; anticipated concerns (providers only); and job satisfaction (providers only). Within these categories we describe and compare patients’ and providers’ experiences and perceptions of barriers and facilitators following KFRE implementation. Illustrative quotes are provided in Table 3; an expanded table of quotes and a summary of pre- and post-implementation category relationships and definitions are available in the supplementary material (Additional files 4 and 5).

**Table 3:** Selected illustrative quotations

Category	Illustrative Quotations
Patients and Providers	
Targeted care	Patient: We have been discussing the issue for 6 months or once a year for awhile and I assumed when they gave me the letter saying that I was now below the 5% threshold for likely dialysis in the next 2 years and would not be using the clinic facilities completely but simply meeting with [nephrologist] that would probably would go to the 1 year [appointment] and when we talked about it this February we decided that would be frequent enough and he is available if something happens. If nothing happens then he can spend the time looking after those who still have more need than I do. It was included in the letter that said that due to this grading, I would no longer make use of the kidney clinic service, but only the nephrologist. Interviewer: and what did you think of that letter? Patient: I thought it was very appropriate. We need to conserve our resources and use them where they are needed and I think at this point in time, that was something I didn’t need, so I know there’s always more people, there’s more demand than we can meet. To make sure that people actually get appropriate care, that the sick people are being seen where they should be seen. (nephrologist) I think yearly is fine you know, as long as I’m stable there’s no point in me going in every 3 months, so she can tell me that ‘yup, everything is stable’. (patient) It’s changed my practice … there are some people that I probably wouldn’t have referred previously because I was using a GFR of less than 30 with evidence of decline and complications. So, now the people who might have a little bit higher GFR but are at high-risk, I’m referring those people. (nephrologist)
Access to resources outside the multidisciplinary CKD clinic	I don’t really have to access [allied health professionals] other than going to my pharmacist … as far as dietitians, when I do see one, they are pretty helpful but … everything is pretty stable, I’m up to yearly visits with [my nephrologist] and nothing has really changed. (patient) Patient: When I do my bloodwork in between [general nephrology appointments] I get it [the results] from my family doctor rather than from the clinic. Interviewer: are you comfortable talking about your CKD with your family doctor? Patient: Yup, no problem at all, he’s well aware of it and has kept up even when I was doing the clinic. If there were any questions that I had in-between I was able to get him to look them up and deal with them. I did rely on my nurse [case] manager quite a bit. She was my go-to person … I don’t have that anymore, but [my nephrologist] has made sure I have a phone number to phone if I need something, but it’s not as direct as dealing with [the nurse case manager]. (patient) I haven’t discharged [low-risk patients] from [the CKD clinic] back to their primary care provider because I have absolutely no confidence that the right amount of supervision and care will be applied to those patients. (nephrologist) I think really the only thing would be that, I mean even though patients that have higher GFRs and do fall in that KFRE where they are low risk...they still do have questions, I think they can still sometimes use guidance you know, for concerns around their kidneys and things like that, so you know the only thing is that they don’t really have that kind of easy accessibility to a nurse or to someone to call about their kidney issues, or you know that kind of thing. So, you know the only thing is, then they don’t have as many resources. (nurse) I’ve really tried to direct them to the [primary care networks] PCNs … if someone is at low risk of progression to kidney failure, why are we using resources that are in limited supply? (nephrologist) There needs to be a general focus in the CKD clinic on respect for the work of family physicians. Too often I hear negative comments that may serve to undermine our family medicine colleagues. (nephrologist)
Self-efficacy	I do a pretty good job of keeping track of what I should eat and should not eat...and it seems to reflect in my [bloodwork] levels … it’s not really a high rate of kidney function but it stays the same. So this charting of it helps me know what to eat and what not to eat. (patient) Going to a GP [General Practitioner] just to get lab results seems a little bit excessive... [A patient portal to access lab results] would be marvelous. (patient) I would use [a patient portal to access lab results], absolutely, that way I don’t have to wait and see [my nephrologist] … because then I can do things before I see [my nephrologist] (patient). They will allow [online] access to bloodwork at one point... I think that will help because most of [patients] just want to know their numbers, they don’t ask us about anything and just want to know the numbers. (nurse)
Patient reassurance and reduced stress	Having that equation and knowing that the chances of me going into kidney failure in the next 10 years is extremely low was quite reassuring. (patient) It actually really helps, really reassuring the patient and making sure that they are aware of what their risk is and I think it paints a really good picture and it’s a really great educational tool. (nephrologist) I miss not talking to them [the nurses], but I’m glad I don’t have to do it because it’s difficult for me to get there...I’m not unhappy about not going. (patient) Some of them are quite happy. Some of them are really happy to back to their GP, it’s just one less appointment that you know they don’t have to come as often. They feel like...they are getting better somehow and they are happy with it. (nurse)
Transition process for low-risk patients	I would say about 90% of them [patients] have proven not to have any problems [with the discharge from the kidney clinic], but I do have patients that call and say, well ‘my medication’s run out and I need to renew and I don’t know what to do’. Previously, I had always contacted the pharmacy and make sure that any renewals get faxed to the nephrologist office to get renewed. (nurse) It came as a surprise to me, I wasn’t really expecting it, but I guess a little more information or explanation at that time might have been a little bit helpful. (patient) For me, it’s just spending a lot of time with them and making sure that they really understand that their risk is low and that they are not being abandoned … It’s really the reassurance and, for certain patients, if they are still anxious and really upset … I usually just offer them either slightly more frequent follow up … for the most part there hasn’t been any major issues. (nephrologist) I think a lot of doctors are supporting your tools and they are doing it really good and as soon as it’s [the KFRE is] less than 10% we discharge them [from the kidney clinic], but some doctors have a tendency to keep their patients with us because I think it’s easier for them because we provide support for them and they don’t have to, they have less to do, right and so yeah sometimes it’s not the best reason [to keep the patient in the kidney clinic]. (nurse)
Providers only	
Anticipated concerns	The nurses give the patients a confidence and an education and supervision that keeps them focused on doing what is, at least to the current literature, correct which is diet management, blood pressure management, medication adherence. (nephrologist) I don’t know if the tool is predicting those high-risk patients who tank right away … I’m just sometimes a bit concerned about that. But, it’s hard for me to say if it was the same before [KFRE implementation]. (nurse)
Job satisfaction	All my easy patients are not there anymore [at the multidisciplinary clinic]...I have to spend more time prepping my chart...I do feel that the workload has gone up a little bit … but that’s the right thing to do, that’s why they are there. (nephrologist) I don’t think implementing the KFRE has dramatically impacted [nurses] workload. It has dropped their numbers substantially, yes. But, most of the patients they have discharged from their caseload … were the ones that didn’t call them anyway … it’s the really acutely ill patients that have been left on the caseload, so you know they had 160 [patients] and now they have 120 or 130 but they are the really sick 120–130 that you’re managing 80% of the time anyway and you know the [caseload] numbers look great, but it’s not indicative of the workload...the workload is still high. (nurse) Lower caseload for case management and that gives us more time to focus on high-risk patients. (nurse) We never have full staff. We always have to cover one another, so if we have three staff that phone in sick, [the] remaining three nurses have to double up their workload. (nurse)

#### Content categories identified by patients and providers

***Targeted care*** *: this category includes reference to personalized medicine, targeting care based on individual needs, ensuring patients are seen in the most appropriate setting, and sustainability of risk-based model of care.*

Both patients and providers reported that using the KFRE to target care to patients most likely to progress was a key strength. Providers indicated that the KFRE was a ‘useful tool’ to help utilize clinic resources optimally, limit ‘inappropriate referrals’, and ensure maximum benefit to patients in need of targeted intervention. Patients discharged from multidisciplinary care following KFRE implementation echoed this sentiment, indicating that as long as they are ‘stable’, ‘there’s no point’ going to the clinic more often, or accessing more resources. Some providers stated they changed their referral practice as a result of the KFRE implementation, suggesting they may have more confidence in their ability to identify high-risk patients.

***Access to resources outside of the multidisciplinary clinic:*** *this category includes examples of resources that may or may not exist outside of the multidisciplinary clinics and patient/provider awareness, need (or lack of need) and comfort accessing those resources.*

Most patients reported they had adequate access to reliable resources outside of the clinic to help manage their CKD including primary care providers, nurse practitioners, dietitians, and pharmacists. Moreover, many patients reported that they did not ‘really have to access’ additional services because their disease was ‘stable’. However, some nurses indicated that patients discharged from the CKD clinic were still phoning them, primarily to inquire about blood work results, suggesting that ‘patients are calling us back because they don’t have the same support anymore’. Although patients reported that they ‘did rely on their nurse case managers’ in the past, they did not perceive a substantial impact to their care following discharge from the multidisciplinary clinic, suggesting they are able to receive the care they need.

A few providers expressed concern about access to preventive education and adequate monitoring outside of the multidisciplinary clinic; one provider in particular indicated they were reluctant to discharge low-risk patients because they were not ‘confident’ that the ‘right amount of supervision and care’ would be provided. However, several providers reported there were many resources in the community, such as primary care networks and nutrition classes/resources. Some providers suggested that the perception of inadequate monitoring in the community might be due to a lack of ‘respect’ toward primary care in general.

***Self-efficacy:*** *this category includes patient and provider comments on, or examples of, self-management and health literacy following implementation of risk-based care.*

Many of the low-risk patients demonstrated good self-management and self-efficacy, reporting that they ‘keep track of’ their diet and lab information and know how to manage and help slow progression of their CKD. However, these patients also reported that one aspect of care they missed was timely access to their laboratory test results, commenting that the ability to access those values on their own would be ‘marvellous’ so they ‘don’t have to wait to see’ their nephrologist and can ‘do things before’ their appointment to manage their CKD. Similarly, nurses also indicated that access to laboratory test results was one of the primary reasons patients called them and that an online patient portal to access bloodwork would be helpful because most patients ‘don’t ask us about anything [when they call]’, ‘they just want to know their numbers’.

***Patient reassurance and reduced stress*** *: this category includes references to reassurance gained from knowing the KFR value and patient or provider comments on patient emotional well-being following implementation of the risk-based approach.*

Most patients indicated that knowing their KFRE value was very ‘reassuring’. Providers also articulated the relief experienced by patients, particularly when the patient’s risk of kidney failure was low, and indicated that the KFRE was a ‘great educational tool’ to communicate individual risk. Additionally, both patients and providers reported that some patients were quite relieved to have fewer appointments when transitioned back to general nephrology or primary care, particularly when their CKD was stable, and that they were ‘not unhappy about not going’. Several providers echoed this sentiment, suggesting many patients ‘are really happy to go back to their GP [general practitioner], it’s just one less appointment … they feel like they are getting better somehow and they are happy with it’.

***Transition process for low-risk patients:*** *this category includes comments related to the transition process, challenges experienced, and reluctance to discharge lower-risk patients.*

Providers identified several challenges related to the implementation, particularly relating to discharge of prevalent multidisciplinary clinic patients to general nephrology. Most issues were administrative, related to relocation of patient medical charts, updating medication renewals, changing the bloodwork requisition and frequency, and general communication specific to this transition. A few patients indicated they would have appreciated ‘more information or explanation’ to process the transition. Other patients, although recognizing they were stable and may not require multidisciplinary care, still expressed disappointment about not having access to their nurse anymore, commenting that ‘more access to a nurse would probably be a little more helpful … even though … nothing has really changed in three years’.

#### Content categories identified by providers only

***Anticipated concerns*** **:** *this category includes perceived concerns about the KFRE’s predictive ability and potential, perceived long-term risks for low-risk patients due to lost access to a nurse case manager and multidisciplinary resources.*

Some providers were concerned about long-term outcomes for low-risk CKD patients who will not receive ‘education and supervision’ from the multidisciplinary team to keep them ‘focused’ on slowing progression. These concerns were amplified by a perception that a low KFRE value might provide a false sense of security for patients and their community healthcare providers. A few providers also expressed concern about those patients who might ‘tank right away’, and whether the KFRE would identify a rapidly progressing CKD patient in time. Patients did not express specific concerns about future progression of their kidney disease.

***Job satisfaction:*** *this category includes references to workload changes and overall job satisfaction following implementation of the risk-based approach.*

In general, nephrologists reported that although the KFRE created additional ‘workload’ for them, they acknowledged that it was ‘the right thing to do’. In contrast, nurses and other staff indicated that the KFRE had a slight positive impact, or no noticeable impact, on their workload and job satisfaction. Many nurses reported they were grateful for ‘more time to focus on high-risk patients’; however, some nurses felt that as a result of the KFRE their caseload was more acute and, since patients who were discharged required minimal monitoring, their workload was relatively unchanged. Nurses’ workload, and possibly job satisfaction, may have been impacted by a number of concurrent staffing issues that arose during the post-implementation study period; several staff went on leave (medical and maternity) and as a result, caseloads were re-distributed.

### Quantitative component

#### Patient care experience

Overall, 176 patients completed the care experience survey in the pre-implementation phase and 237 in the post-implementation phase. Most participants in both phases were male, married, > 65 years of age, and reported their health status as “Fair” or “Good” (Table 1).

**Fig. 2:**
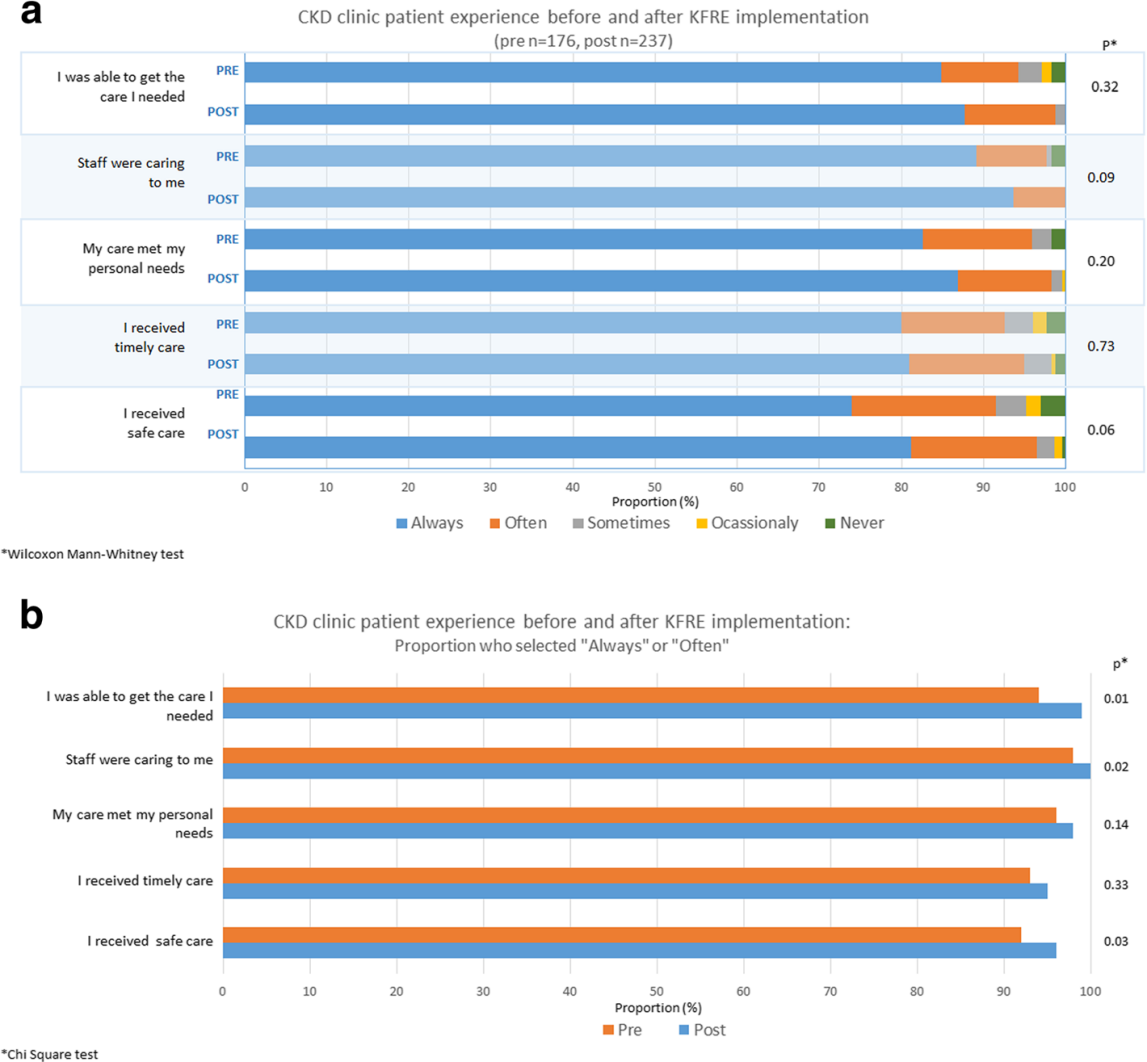
**a** Chronic Kidney Disease (CKD) multidisciplinary clinic patient experience before and after KFRE implementation **b** Proportion of respondents who selected “Always” or “Often”

The majority of patients were satisfied with their care in both the pre- and post-implementation periods, with no significant differences in any of the care experience domains when analyzing all five categories (Fig. 2a). When considering the responses “Always” and “Often” together versus not (Fig. 2b), there were slight, but statistically significant, improvements in three of five domains in the post-implementation period: access to care (*p* = 0.01), caring staff (*p* = 0.02), and safety of care (*p* = 0.03).

#### Provider job satisfaction

Of the 75 healthcare providers, 40 (53%) completed the workplace satisfaction survey in the pre-implementation phase and 33 (44%) in the post-implementation phase. The majority of respondents were female with > 10 years experience in their profession and > 5 years working at the CKD multidisciplinary clinic (Table 2).

**Fig. 3:**
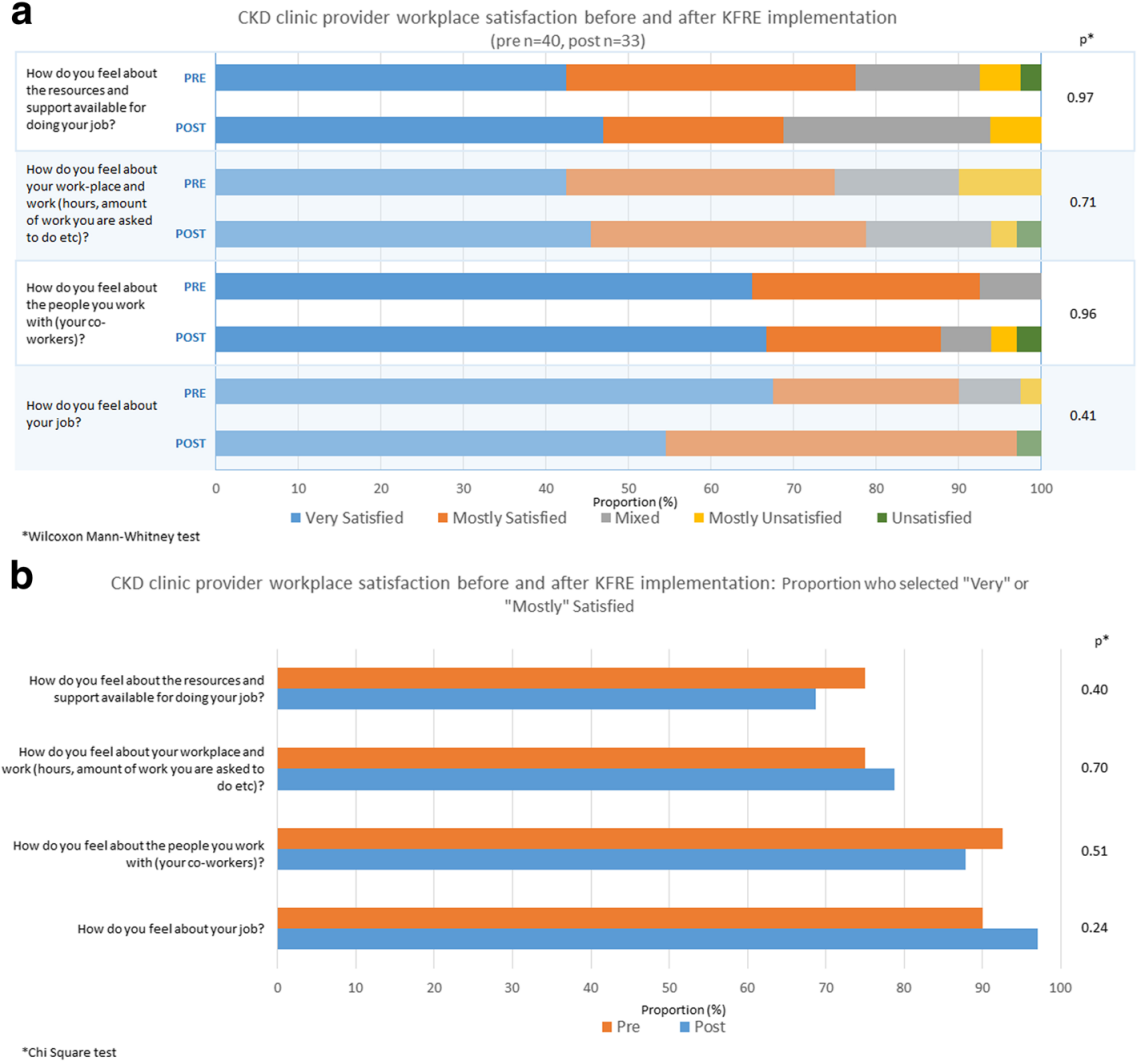
**a** Chronic Kidney Disease (CKD) multidisciplinary clinic healthcare provider satisfaction before and after KFRE implementation **b** Proportion of respondents who selected “Very” or “Mostly”

There were no significant differences in workplace satisfaction items pre- and post-implementation of the KFRE, with the majority of participants reporting they were satisfied (Fig. 3a). When considering “Very” and “Mostly” satisfied responses together (versus mixed or unsatisfied responses) for each scale item there were no significant differences (Fig. 3b).

## Discussion

In this mixed methods study, patients and healthcare providers described benefits and challenges associated with using the KFRE to stratify CKD patients to care providers based on their risk of kidney failure. Overall, patients and providers felt that a KFRE-based approach improved sustainability and quality of the multidisciplinary clinics by targeting care to patients at highest risk; however, some providers expressed concern that care for low risk CKD patients may be of lower quality, and that access to care may be inadequate. There were no significant differences in job satisfaction for healthcare providers, whereas patient results suggested slight improvement in care experience following KFRE implementation.

Similar to our pre-implementation study [13], the primary concern identified by healthcare providers regarding use of the KFRE to risk stratify patients for multidisciplinary care was potential for patients with lower risk of kidney failure experiencing more rapid progression over time without multidisciplinary care. These provider concerns may stem from poor perceptions of patient self-efficacy and primary care in general and is consistent with other studies [26–31]. Although challenges related to identification and management of CKD patients in primary care have been reported [2], subsequent strategies to build CKD awareness, education, and access to specialist advice, such as an online CKD clinical pathway [32–34] and electronic access to specialist advice [35], have been implemented to build healthcare provider capacity to care for CKD patients in the community.

In contrast, low-risk patients who were transitioned out of the multidisciplinary care clinics to general nephrology care reported they had adequate access to, and confidence in, their community-based primary care physicians, dietitians and pharmacists, and did not perceive a substantial impact to their care. These patients described a number of strategies they used to manage their CKD, such as diet and clinical lab results tracking. The threshold we used to consider multidisciplinary care was relatively low (≥ 10% risk of kidney failure in 2 years), and the majority of patients discharged from the clinic continue to be managed in general nephrology. Our ongoing prospective evaluation using our provincial data sources [36] will address this concern by assessing the risk of kidney failure for patients not followed in the multidisciplinary care clinic. Additionally, a self-management eHealth tool [37], co-developed with patients and providers, and a provincial patient portal [38] to access individual medical information online, are both in development. These tools will complement existing resources, support patients, and encourage self-efficacy.

A number of providers remained concerned regarding the KFRE’s accuracy and consequent long-term patient outcomes. Although some studies suggest multidisciplinary CKD care leads to improved patient outcomes, decreased hospitalizations, and reduced healthcare costs in pre-dialysis CKD patients [39–42], a recent systematic review [12] concluded that most published studies demonstrate a high risk of bias. While there was improvement in blood pressure control and prescribing practices in nurse-led and pharmacist-led care models, they found limited evidence of a significant effect on most outcomes [12]. Moreover, previous research indicates that CKD trajectories in the 2 years preceding dialysis are substantially heterogeneous [43] and the cost-effectiveness of multidisciplinary care may be greatest in younger patients and in those with higher urine albumin levels [44]. As a result, further targeting multidisciplinary clinic services based on individual characteristics has been suggested to optimize patient outcomes and resource use [45]. Assessment of long-term outcomes (hospitalizations, death, modality choice, kidney transplantation, physician visits, and process-based indicators of appropriate CKD care) using provincial administrative data is the focus of the next phase of this study [14].

Strengths of this study were inclusion of both patient and provider perspectives, and the integration of qualitative and quantitative components. However, there are limitations that should be recognized when interpreting the results. We acknowledge that the qualitative component of this study included a larger number of healthcare providers than patients; however, data saturation was achieved in both groups despite differences in sample size. Most of the patients who participated in the interviews had been exposed to multidisciplinary care for > 5 years prior to discharge and may be inherently more proactive, which may have impacted our findings relating to self-efficacy and may limit transferability of these findings to patients with lesser multidisciplinary clinic experience. Moreover, interviewed patients had been discharged from multidisciplinary care within the previous 12 months, following implementation of the risk-based approach, and therefore their described experiences and perceptions are reflective of this limited period. The pre- and post-implementation surveys were anonymous, so it was not possible to determine if participants completed both surveys to pair survey responses, or to determine the patient survey response rates. Finally, these results are based on a single Canadian centre, which may limit generalizability to other settings.

## Conclusion

In summary, evaluation of risk-based models to guide triage and care delivery for CKD patients has been limited. Our findings suggest patients and healthcare providers perceive that a KFRE risk-based approach may improve sustainability and focus of team-based multidisciplinary CKD clinics by targeting patients at highest risk, with survey results suggesting no difference in healthcare provider job satisfaction and potential slight improvement in patient care experience. However, some providers remain concerned regarding the KFRE’s accuracy and consequent long-term patient outcomes for lower-risk patients risk-stratified to receive care in general nephrology only. This study is one component of a multiphase study evaluating use of a KFRE-based approach to guide CKD care. In the final phase, provincial administrative data will be used to prospectively evaluate the effectiveness of this risk-based approach on important patient care and clinical outcomes.

## Additional files

Additional file 1: Patient Interview Questions: a list of the interview questions included in the semi-structured patient interviews. (PDF 305 kb)

Additional file 2: Provider Interview Questions: a list of the interview questions included in the semi-structured healthcare provider interviews. (PDF 272 kb)

Additional file 3: Provider Open Ended Survey Questions: a list of the open-ended survey questions that were included in qualitative content analysis. (PDF 236 kb)

Additional file 4: Patient and Provider Exemplary Quotes: expanded table of patient and healthcare quotations encompassing each described content category. (PDF 129 kb)

Additional file 5: Pre Post Categories Summary: A summary of the pre- and post-implementation content categories reflecting our directed content analysis approach to the data. (PDF 72 kb)

## Abbreviations

CEFIT: Care experience feedback improvement tool
CKD: Chronic kidney disease
KFRE: Kidney failure risk equation
