# Supplementary material for: Patient and provider experience and perspectives of a risk-based approach to multidisciplinary chronic kidney disease care: a mixed methods study
Source: BMC Nephrol. 2019 Mar 29;20:110. doi: 10.1186/s12882-019-1269-2 (PMC6440153; doi:10.1186/s12882-019-1269-2)
Supplement: Supplementary file 1 — Patient Interview Questions: a list of the interview questions included in the semi-structured patient interviews. (PDF 305 kb) [file 12882_2019_1269_MOESM1_ESM.pdf]

Additional File 1. Patient Interview Questions

QUESTIONS:

Note: CKD = chronic kidney disease; MDC = multidisciplinary clinic; NCM = Nurse case manager

About a year ago, there were some changes made to the MDC. We are interested in hearing about your care experience over the last year. When answering these questions, please reflect on the care you have received in the last year only.

As I understand, in the past you had a nurse manager as a part of your care team, but that has changed recently and now you see your Nephrologist only during your clinic visit, is that right?

1. How has it been going since this change?
2. Where do you see your Nephrologist?
3. Thinking about your last visit to your nephrologist, after the change, what has it been like for you when you visit your nephrologist?
    - Have you noticed any changes in your care since the change (either positive or negative)?
    - Have you seen any allied health providers at these visits (eg. dietitian, pharmacist, social worker)? If so,
        - How are the appointments going?
        - Do you schedule a separate visit for these appointments?
        - How do you feel about receiving care from your nephrologist and only visiting allied healthcare providers if/when necessary?
    - Is there anything you would like to see done differently?
    - Are you using different services or resources now to manage your CKD that you didn't before? If so, which ones
        - Do you use the internet to find resources?
        - Talk with peers?
        - Community health care providers?
    - What could help you manage your CKD?
        - Peer support group?
        - Online access to your medical and/or lab information?
4. Since the change, how do you feel about communication with your Nephrologist?
    - Is the information you receive clear?
    - Is there anything that can be improved?
5. Are there any issues or problems that have arisen for you since the change?
6. How do you feel about the amount of time spent at the CKD clinic during your visit?
    - Has it changed since the transition?
    - # of appointments, duration of appointments?

7. Are you aware of the Kidney Failure Risk Equation, or any risk tools used to better understand your risk of developing kidney failure, or requiring dialysis? If so,
    - Is knowing your risk of kidney failure meaningful to you? If so, how?
    - Did you receive a print out with your KFR from your Nephrologist? If yes:
        - What did you think of it?
        - Anything you'd like to see added or changes?

8. Do you see your family physician for your CKD? If so,
    - How do you feel about the CKD care you receive from your family physician?
    - Are there any challenges related to care for your CKD by your family doctor?
    - How do you feel about communication with your family physician for your CKD care?
    - Is the information you receive clear?
    - Is there anything that could be improved? If no, why/why not?

9. Is there anything I didn't ask you that you think I should have? Any other comments or suggestions?
