# Supplementary material for: Patient and provider experience and perspectives of a risk-based approach to multidisciplinary chronic kidney disease care: a mixed methods study
Source: BMC Nephrol. 2019 Mar 29;20:110. doi: 10.1186/s12882-019-1269-2 (PMC6440153; doi:10.1186/s12882-019-1269-2)
Supplement: Supplementary file 2 — Provider Interview Questions: a list of the interview questions included in the semi-structured healthcare provider interviews. (PDF 272 kb) [file 12882_2019_1269_MOESM2_ESM.pdf]

Additional File 2. Healthcare Provider Interview Questions

QUESTIONS:

Note: CKD = chronic kidney disease; MDC = multidisciplinary clinic; KFRE = Kidney Failure Risk Equation

Last Spring, in April 2017, the kidney failure risk equation (KFRE) was implemented in the multidisciplinary clinics to guide clinic eligibility criteria. Patients with a KFRE or ≥10% (or eGFR ≤15) were recommended as eligible for the multidisciplinary clinic, and patients with KFRE <10% were recommended to be followed in general nephrology only. We are interested in hearing about your experiences since the KFRE was implemented, when answering these questions please reflect on the care you have delivered since April 2017 only.

1. How has it been going?
    - Have you run into any problems?
        - Charting for General Nephrology patients?
        - Communication?
        - Ordering labs or other tests?
        - Ideas to improve
    - What are some of the benefits?
    - Have you transitioned patients from the CKD MDC to general nephrology?
        - How did it go?
        - How have patients responded?
        - What barriers/facilitators have you experienced transitioning patients from the MDC to General Nephrology?
        - How do you feel about the KFRE as a: stratification tool? An educational tool?
        - Did you utilize the "discharge" letter or "Your Results" Handout? Why/Why not? Was it helpful?
        - How do you feel about the resources available to general nephrology patients to help manage their CKD? What additional resources are needed?
        - Is there anything else we could provide to help with this transition (for patients and/or providers)?
        - Is there anything you'd like to see done differently?
    - How do you feel this new model impacts your workload?
        - Is it better, worse, the same? How so/explain?
    - How do you feel this new model impacts patient care?
        - Is it better, worse, the same? How so/explain?
    - Is the KFRE tool easy to use? Any suggestions for improvement?

2. Any other suggestions or comments?
