# Supplementary material for: Patient and provider experience and perspectives of a risk-based approach to multidisciplinary chronic kidney disease care: a mixed methods study
Source: BMC Nephrol. 2019 Mar 29;20:110. doi: 10.1186/s12882-019-1269-2 (PMC6440153; doi:10.1186/s12882-019-1269-2)
Supplement: Supplementary file 3 — Provider Open Ended Survey Questions: a list of the open-ended survey questions that were included in qualitative content analysis. (PDF 236 kb) [file 12882_2019_1269_MOESM3_ESM.pdf]

Additional File 3. Healthcare Provider Open-ended Survey Questions

QUESTIONS:

1. Reflecting on the Kidney Failure Risk Equation implementation in the CKD multidisciplinary clinics:

   a. What have been the greatest benefits?

   b. What have been the greatest challenges?

   c. What barriers or facilitators have you experienced transitioning low-risk patients back to general nephrology?

   d. Do you have any additional comments?
