# Supplementary material for: Patient and provider experience and perspectives of a risk-based approach to multidisciplinary chronic kidney disease care: a mixed methods study
Source: BMC Nephrol. 2019 Mar 29;20:110. doi: 10.1186/s12882-019-1269-2 (PMC6440153; doi:10.1186/s12882-019-1269-2)
Supplement: Supplementary file 4 — Patient and Provider Exemplary Quotes: expanded table of patient and healthcare quotations encompassing each described content category. (PDF 129 kb) [file 12882_2019_1269_MOESM4_ESM.pdf]

Appendix 4 Post KFRE Implementation Exemplary Quotations

Category		Healthcare Providers	Role	Mode	Pt ID
Targeted care	•	The KFRE is a helpful tool when screening and prioritizing patients.	Allied Health/Other	Survey	4
	•	Nurses can concentrate their time and efforts to manage patients who require the most kidney care	Nurse	Survey	19
	•	Nurses are able to see their own patients at clinic in a manageable time slot	Nurse	Survey	19
	•	Discharging kidney patients who are at lower risk, and allowing more time for patients with higher risks	Nurse	Survey	21
	•	Our management can be focused on the patient's who we feel need it most, those nearing ESRD	Nurse	Survey	22
	•	It has limited most of the inappropriate referrals to CKD [clinic].	Nurse	Survey	25
	•	In terms of referring there [the CKD clinic] it's changed my practice because I look and there are some people that I probably wouldn't have referred previously because I was using a GFR of less than 30 with evidence of decline and complications. So, now the people who might have a little bit higher GFR but are at high risk, I'm referring those people.	Nephrologist	Interview	401
	•	That whole thing about directing the care to the need, instead of giving everyone the same thing, making sure that you know, if someone's numbers look great and their kidney function is stable, they don't necessarily need to see a dietitian, whereas someone who's got repeated episodes of volume overload getting admitted to hospital they need a lot more attention.	Nephrologist	Interview	401
	•	I think we are doing the right thing. We are focusing our effort on patients who really need, who more likely need our care	Nephrologist	Interview	406
	•	I'm assuming there's less [fewer] patients in the kidney clinic, the multidisciplinary clinic, that I think they are able to attend to the existing population better and they are able to accept new patients faster.	Nephrologist	Interview	407
	•	I think it's a useful tool to try and offload a clinic that is already in really high demand and under stress. It's a really important service having these multidisciplinary clinics and you want to make sure that the right people are actually receiving the care get the biggest bang for your buck. So I think using these types of tools to try and stratify who gets referred and sent over to these high demand, high resource clinics is good and appropriate to make sure that people actually get appropriate care that the sick people are being seen where they should be seen and then the less sick people are followed elsewhere.	Nephrologist	Interview	418
	•	In our resource scarce environment you need to focus our efforts on people who are going to benefit the most and who are at the highest risk and I think this tool and the way we stratify for this clinic is a good way of addressing that making sure the sickest people get the most, best care.	Nephrologist	Interview	418
	•	We follow high risk patients and we are happy to devote our time to those high risk patients because they need us. What we didn't like was those forty, those GFRs of 42, where we see once in every 18 months, we are wondering why they are on our caseload and you can't argue that it's a workload issue in the sense where, well I'm not doing much for them anyway, they only go for bloodwork every 3-4 months; however, my point, our point as a nurse, frontline nurse saying, well we still have to look at their bloodwork in three months time, right? And they are on our caseload they are still our responsibility...so that creates an unnecessary workload even though it's minimal, the point is that, why are they on my caseload when I'm not really doing much for them, right? ...I'm not impacting their care in any way because their blood pressure is stable, their GFR is stable meaning the family doctor or the kidney doctor, the nephrologist, can manage them without me.	Nurse	Interview	419
	•	More appropriate use of CKD clinic resources for patients that are at high risk of progressing	Nephrologist	Survey	2
	•	Streamlining and standardizing referrals. Everyone follows the same rules for referring	Nephrologist	Survey	5
	•	Better directed focus of care to those who require frequent encounters with follow ups and guidance through telephone call follow ups, clinics.	Nurse	Survey	18
	•	It has limited most of the inappropriate referrals to CKD	Nurse	Survey	25
		Better allocation of limited resources	Nephrologist	Survey	9
	•	An empiric tool for level of care. Makes one think of 'Does this person need mutli-discplinary care'	Nephrologist	Survey	13
Patient Reassurance and reduced stress	•	I think it's the relief for the patients. Like some of them just trot out of here, 'I don't need to be followed so much anymore'.	Allied Health/Other	Interview	412
	•	Personally I haven't heard anything about patients not liking it because, from their perspective, they still come to see me at that same place and really they are actually reassured that they don't need to see the nurse because they are better	Nephrologist	Interview	406
	•	It actually really helps like really reassuring the patient and making sure that they are aware of what their risk is and I think it paints a really good picture and it's a really great educational tool	Nephrologist	Interview	407

Appendix 4 Post KFRE Implementation Exemplary Quotations

Category	Healthcare Providers	Role	Mode	Pt ID
Patient reassurance and reduced stress	• I would say staff satisfaction is definitely higher...in terms of the workload, managing their caseload.	Nurse	Interview	419
	• Patients seem quite excited to know that they no longer require such intensive care.	Allied Health/Other	Survey	6
	• I find it most helpful to reassure patients. I will see someone with a GFR of 40 or even 35 and they are young and they are really worried that they are going to need dialysis or end up with kidney failure, so I’ve found it most useful to reassure the low risk patients	Nephrologist	Interview	418
	• Some of them are quite happy. Some of them are really happy to back to their GP, it’s just one less appointment that you know they don’t have to come as often. They feel like...they are getting better some how and they are happy with it	Nurse	Interview	420
Access to resources outside of the CKD clinic	• I'm pretty sure that a lot of them, if they are stable enough and they leave the kidney clinic, they continue to follow the kidney diet and if they have diabetes most of them are followed by the community diabetes centre or followed by their GP, and if they are followed by the community diabetic centre, there is also nutritional information provided...I have patients even now that I’m managing and instead of following up with our diabetes nurses they are followed by the diabetic centre with nurses and dietitians	Nurse	Interview	422
	• I don’t think our social worker resources should be going to like filling out disability forms and helping with disability forms in someone who is really low risk. It should go back to their family doctor	Nephrologist	Interview	401
	• There needs to be a general focus in the CKD clinic on respect for the work of family physicians. Too often I hear negative comments that may serve to undermine our family medicine colleagues	Nephrologist	Survey	2
	• Patients became used to the Multidisciplinary approach this program had provided, a very positive approach, that they are unable to achieve in their GP offices due to lack of clinic time given to them outside this program	Nurse	Survey	18
	• I’ve really tried to direct them to the PCNs [Primary Care Networks] and what’s available...PCNs have lots of great resources, so if someone is at low risk of progression to kidney failure, why are we using resources that are in limited supply...a lot of these [patient's] don’t need to be followed by me.	Nephrologist	Interview	401
	• I haven’t discharged them from Sheldon Chumir back to their primary care provider because I have absolutely no confidence that the right amount of supervision and care will be applied to those [low risk] patients	Nephrologist	Interview	408
	• We still need better supports and access to supports for patients 'up-stream' to help them avoid needing our clinic in the future.	Allied Health/Other	Survey	3
	• Lots of resources are going into primary care, patients want their care closer to home and so I’ve been really trying to figure out, you know, is their someone who can deliver this service through their primary care provider, so I haven’t really had any issues there	Nephrologist	Interview	401
	• I think sometimes, well people don’t know exactly where to go then after [discharge from the kidney clinic] if they have nutrition concerns. Because even if they are not part of our clinic, they can always be referred to our general nephrology dietitian. There’s also dietitians in the Alberta Healthy Living program they can access.	Allied Health/Other	Interview	425
	• There was only one time that it was unusual that I thought it’s a good thing I went to see this person because he, his KFRE was like 2 or 3% so he was very stable, he was going to be discharged. I happened to go in to see him just because I hadn’t seen him for a year or something...we started talking and he said his wife had passed away and his daughter was there and another sibling had passed away and so now they were kind of on their own and not the people that were used to cooking they were like, 'I need some help'. So, it was really good that I had went in. And, you never would have known looking at that number that he needed some assistance. So that’s the only thing, I mean and unless they bring it up with the doctor then the doctor won't know to ask us to see them.	Allied Health/Other	Interview	425
	• Some patients are still high risk based on other co-morbidities or psychosocial situation and the worry is they are not being as fully supported as they could be. How do we provide adequate risk education, diet, blood pressure, blood sugar, fluid management to those patients who do not qualify for CKD management? group classes? online education? making this education mandatory for general nephrology patients who are not high risk?	Nurse	Survey	23
	• I encourage my patients to tap into the Living Well program to manage their chronic disease. Not only kidneys, there are other things too...they just need the website or the phone to pick the phone to make an appointment and most of those patients, you have no idea how appreciative just that piece of information [is]...what I hear from our patient group, none of the doctors ever mention it, it’s usually the nurses that mention it...for those people that got discharged from our clinic we encourage them to, if they want more information about the kidney health, I encourage them to sign up for the kidney health class.	Nurse	Interview	424

Appendix 4 Post KFRE Implementation Exemplary Quotations

Category	Healthcare Providers	Role	Mode	Pt ID
**Access to resources outside of the CKD clinic**	• All the new patients are asked to come to a CKD nutrition class...but not everybody comes to that and of course patients who are longstanding at the clinic and then get discharged wouldn't have that...people that are in general nephrology can also attend the different programs that we have, right, like the renal community kitchen or the grocery store tours or things like that. So, yeah they are definitely, there could be something that people are provided with if the get discharged if they have questions and one of the websites, actually you can ask a renal dietitian questions...there's a variety of renal dieticians across Canada that take turns answering questions...www.kidneycommunitykitchen.ca.	Allied Health/Other	Interview	425
	• I think really the only thing would be that, I mean even though patients that have higher GFRs and do fall in that KFRE where they are low risk...they still do have questions, I think they can still sometimes use guidance you know, for concerns around their kidneys and things like that, so you know the only thing is that they don't really have that kind of easy accessibility to a nurse or to someone to call about their kidney issues, or you know that kind of thing. So, you know the only thing is, then they don't have as many resources	Nurse	Interview	413
**Self-efficacy**	• They will allow [online] access to the blood work at one point, I don't know when that's going to happen, but I think that will help with that because most of them just want to know the numbers, they don't ask us about anything and just want to know the numbers...So I think that would help.	Nurse	Interview	414
	• Clients who had good knowledge and adherence to CKD instructions on kidney/diabetic diet, esp low salt, restrict potassium, monitor blood pressure, blood sugar, weight, know how to check for edema, know fluid hydration and to adjust for edema, familiar with medications and reason they were prescribed. These are the patients that would do well on discharge	Nurse	Survey	16
**Anticipated concerns**	• You are decanting a problem from one overloaded clinic to another overloaded clinic...or one overloaded booking clerk to another overloaded secretary or booking clerk, so the problem here is resources	Nephrologist	Interview	408
	• It's the new referrals who had not have adequate absorption of education from CKD who would end up returning after a few months	Nurse	Survey	16
	• Patients became used to the Multidisciplinary approach this program had provided, a very positive approach, that they are unable to achieve in their GP offices due to lack of clinic time given to them outside this program	Nurse	Survey	18
	• The general practitioners and frankly many sub-specialists don't share the anxiety that we do that we are going to have to start another patient on dialysis. They think dialysis, well it's free it's there, it's cheap, it's easy, just send them to the nephrologist and let them take care of it, but that anxiety level for me and the patient is a hell of a lot more than what I need to take on. I would rather struggle and fight with the patient about their diet and their blood pressure control and everything else for years beforehand then to deal with a patient on dialysis. A patient on dialysis is really difficult psychological model to wor with. They are angry, they are disappointed, they are depressed...the KFRE will help but not by giving people passive confidence that everything is wonderful, everything is sunny at 2%.	Nephrologist	Interview	408
	• When the problem comes, what do they [the patient's dischaged to general nephrology] need? if their blood pressure is getting higher, what they doing? Ar they waiting? Do they know what to do, to phone their family doctor? Or they just wait until they see their family doctor next, right? [When they were kidney clinic patients] they would just call us and we'd say, "we can wait until the next clinic visit" or "I will [talk to the] doctor and I will get back to you". So, just those I don't know what they are doing, I have no idea	Nurse	Interview	414
	• I don't know if the tool is predicting those high risk patients who tank right away. So if they are like 5% at one point and three months later like 15, 20, 30% they tank like really fast, so yeah I'm just sometimes a bit concerned about that. But, it's hard for me to say if it was the same before.	Nurse	Interview	414
	• If I'm a general practitioner and my nephrologist sends me that report of 2% [kidney failure] risk in the next two years, the general practitioner is going to turn around and say well 2%, he has a 10% chance of dying in an automobile accident in the next two years, actually in the next year according to Canadian statistics, so he's more likely to die in an automobile accident than from his kidney disease, I don't need to see him for two years. In the meantime his blood pressure is taking off and his diet has been forgotten and his proteinuria has increased and nobody knows or cares.	Nephrologist	Interview	408

Appendix 4 Post KFRE Implementation Exemplary Quotations

Category	Healthcare Providers	Role	Mode	Pt ID
Anticipated concerns	• It's the proper care after [discharge from the kidney clinic] and I'm wondering if their progression will get faster because they are outside the clinic or they will stay the same.	Nurse	Interview	414
	• I think you have to wait a little bit, a few months and see how many people are coming back to us. Because those patients that we saw for two, three months and they are discharged to the GP again, or the nephrologist, I'm not sure if they are going to tank again and come back to us right away. So I would like to know how many people are coming back to us or the people we discharge because they were stable for years, now they don't have our support for asking questions and blood pressure and everything. I'm wondering if they are going to come back in a few months.	Nurse	Interview	414
	• The patients are going to be stuck falling through the crack and all of a sudden you are going to see a blip in the number of peritoneal dialysis patient and hemodialysis patients because that's where they are going to end up, as crash-care models	Nephrologist	Interview	408
	• The KRE formula is a good formula, but to use it to exclude people from a better program is false security because in two years or three years that patient is going to come back ignored by the primary care system. There is just not enough capacity or willpower to supervise the patient to get the best possible lon term outcome and when they come back in two or three years by re-referral and it takes six months to get into the system, or more, by that time they've lost so much irrecoverable renal function. Now, what do you do? That patient got left behind and I think that's wrong.	Nephrologist	Interview	408
	• I think overall KFRE is a good tool to evaluate patient's risk but on the other hand, I think some patients who are not qualified at first to be referred to us will benefit from our education to delay the progression of CKD. I found that now, patients are high risk and we tried to help them but sometimes it is already too late to delay the progression	Nurse	Survey	20
	• In terms of prognosis the tool has weaknesses because it's based on a model where the relationships are ideological and not prognosti	Nephrologist	Interview	403
	• KFRE can change very quickly, I question the accuracy	Nephrologist	Survey	14
	• There's hidden value by having nurses involved in the advanced care model, which KFRE would pretty much exclude for a lot of patients	Nephrologist	Interview	408
Transition process for low risk patients	• overall it's going well. I mean the patients that are low risk are very happy to hear that and yeah those patients weren't using a lot of our services, so to tell them they aren't going to have them, isn't a big deal for a lot of them, so it's ok	Nurse	Interview	423
	• just spending a lot of time with them and making sure that they really understand that their risk is low and that they are not being abandoned and once we really make that clear for the most part the patients seem to be ok, it's really the reassurance and you know for certain patients if they are still anxious and really upset about this type of thing or getting back to general nephrology, I usually just offer them either slightly more frequent follow up or even in certain cases even or specific case, I would say, you know I'll give you guys a call just to checkup on to see how things are just to reassure them. For the most part there hasn't been any major issues	Nephrologist	Interview	407
	• Nephrologist: Slowly prolonging follow-up and then discharging them. I've sent people back to their family doctor<br>Interviewer: ok, so when transitioning patients back to general nephrology or to their family care provider, how has that gone from your perspective and from patient's perspective?<br>Nephrologist: I found it's gone fine from the patient perspective. I feel like the staff have struggled with it...it's interesting because they talk a lot about how busy they are, but they seemed to have developed relationships and have a lot of trouble stepping away and recognizing that they should place their resources towards higher need patients	Nephrologist	Interview	401
	• I noticed there's been some discharges and there was no one that comes to mind that I felt strongly needed our intervention or needed a dietician that was discharged where they couldn't get access to a better dietitian or another dietician...[I] never that I felt like they really needed more information on the renal diet I guess. They might have had other nutrition concerns, but it could be addressed with other dieticians.	Allied Health/Other	Interview	426
	• No real barriers have been experienced however we do get ongoing calls from pt's that have been discharged as they do not realize that they are to contact the Nephrologists office after discharge	Nurse	Survey	17
	• Some pts still call case manager for various questions re: lab requisitions, appts or locations or sometimes just want to talk to someone for encouragement.	Nurse	Survey	19
	• A lot of patients are calling us back (RNs) because they don't have the same support anymore	Nurse	Survey	20
	• I have discharged several patients based on the KFRE and then find it very difficult to transition them back into my regular UCMC clinic - am worried about losing charts/ follow up appointments etc.	Nephrologist	Survey	8

Appendix 4 Post KFRE Implementation Exemplary Quotations

Category	Healthcare Providers	Role	Mode	Pt ID
**Transition process for low risk patients**	• [Discharging patients back to general nephrology] went fine, really the only thing is we would get the odd phone call from a patient that was discharged you know, asking, you know just calling to ask something or ask about a bloodwork req or something like that, and you know, and then we would kind of have to say, well actually we are no longer your nurse, so any questions, or you know if you need a bloodwork req, whatever, you need to phone the nephrologist's office. So we've gotten the odd phone call. I wouldn't say it's been crazy but the odd phone call.	Nurse	Interview	413
	• The ones who have been with us a long time, they have trouble letting us go, so they call us back all the time, right, so we try to let them know that we are not their case manager anymore, but even if you say that, you give the doctor's office number, the nephrologist or anything, they call you back the next month...there are a few patients like this, but most of them are doing pretty good...sometimes they ask for a little bit of advice from us, like the next month [after] they have been discharged [from the kidney clinic] and stuff and we direct them a bit and after that they don't call back anymore, I think the people who have to get used to having their bloodwork right away...I think it's hard for them to let things go, right. It just, because the doctor won't call them righ away, right. We [the nurse case mamagers]do, so it's kind of harder for them.	Nurse	Interview	414
	• I think a lot of doctors are supporting your tools and they are doing it really good and as soon as it's [the KFRE is] less than 10% we discharge them [from the kidney clinic], but some doctors have a tendency to keep their patients with us because I think it's easier for them because we provide support for them and they don't have to, they have less to do, right and so yeah sometimes it's not the best reason [to keep the patient in the kidney clinic].	Nurse	Interview	414
	• If you are discontinuing those [multidisciplinary] services but keeping them in the same environment, I think it's an easier transition, but I imagine it's a hard transition for patients who are used to having such intensive, involved care, having quick prompt follow-up. you know you call your nurse any time you have a question to all of a sudden having a busy nephrologist who can't talk to you everyday	Nurse	Interview	418
	• I would say about 90% of them [patient's] have proven not to have any problems [with the discharge from the kidney clinic], but I do have patients that cal and say, well "my medications run out and I need to renew and I don't know what to do". Previously, I had always contacted the pharmacy and make sure that any renewals get faxed to the nephrologist office to get renewed and so now if they call me it's not that I am rejecting their call, I'm just saying that next time it happens just tell your pharmacist to fax everything over to the doctor's office and get it renewed. And, so that's another piece of ongoing education.	Nurse	Interview	422
	• There have been some [patient's] that probably, I guess maybe shouldn't have been discharged because they were so attached and are used to calling in. You know, like I had one patient, belongs to a different nurse, but I was in the clinic when she was discharged and she was quite concerned because she loves to know her numbers and her bloodwork and the physician assured her, you can just call my office, I'll give you the numbers. And, as I'm sitting there I'm thinking that's not going to happen, I mean right when she wants them, right, because they are so busy or they are on service or they are on holidays and the secretaries can't give them the information. So she'd call, it happened the very first month she was discharged. She called his office, wanted her numbers, he's not in the office, she called me the same day because she still had my number and said I'd like my numbers. And, I said well the doctor said he'd give them to you, you know give him a day or two at least. I mean he may not be in his office today, [the patient] called me the next day, "he still hasn't gotten back to me". So of course I gave her the numbers, but now she's called me every month for three months in a row, I've got a messag just yesterday, right, because the doctor hasn't called her. I mean it's not realistic for the, I don't know why they've said that because it's not realistic for them, they are too busy to be doing that, right. So, I've tried to have her understand if there was something of concern of course they'd get back to you, bu just to call normal stable lab work. Like I mean we get frustrated doing it...so there are some patients that are having trouble and maybe in her case, you know when I see the physician next I will say, maybe she should be back here.	Nurse	Interview	423
	• The issues is you get on the phone with these patients [discharged from the kidney clinic] and it's not just about their lab work, they've got an issue with this, this and this and now, I'm having to deal with it and I can't account for that time, right, because they are not part of our clinic anymore unfortunately. So, I think, I look back through my caseload of patients that I've discharged, I've only had three different patients of the 15 that have called me back asking various things. I've just had to reinforce like I'm no longer following you care and you know, if this is a kidney issue so please contact the physician's office and gave them the number again. But, it is hard and those were the patients that I was a little more involved with, you know before discharge, so it's just a harder transition for them. I'm sure if it becomes an issue on the physician's end they will probably end up bringing them back [to the kidney clinic] right	Nurse	Interview	423

Appendix 4 Post KFRE Implementation Exemplary Quotations

Category	Healthcare Providers	Role	Mode	Pt ID
Job satisfaction	• I think it's impacted the nurses workload…. [nurses] were at 160 patients per nurse and the last count was 125 or so. So I think that's a huge difference.	Nurse	Interview	419
	• The KFRE did not seem to reduce the amount of patients in my caseload		Survey	1
	• I have discharged several patients only to bring them back again not long after which represents an increase in my workloac	Nephrologist	Survey	8
	• Overall it seems to have been beneficial but I don't know if it has decreased the work load as drastically as we all hoped. Some of the patient's that were discharged were the low maintenance patient's	Nurse	Survey	25
	• I appreciate the time I can spend with my high risk patients instead of the low risk patients that were not using the clinic to its full potentia	Nurse	Survey	21
	• Lower caseload for case management and that gives us more time to focus on high-risk patient:	Nurse	Survey	20
	• One of the best ting done in the CKD clinic in the past 15 years	Nephrologist	Survey	13
	• [An] Increased patient load in my own general nephrology practice (is a challenge]	Nephrologist	Survey	5
	• Interviewer: Do you feel that this model with the KFRE had impacted your workload?<br>Nephrologist: yeah...all my easy patients are not there anymore and that's the right thing to do...I have to spend more time prepping my chart...I do feel that the workload has gone up a little bit just because all I have down there now are more progressive disease patients, but that's the right thing to do, that's wh they are there.	Nephrologist	Interview	406
	• I don't think I changed my practice, I don't know maybe the numbers overall are different, but I didn't have the feeling it was it really materially affected my practice.	Nephrologist	Interview	403
	• I think you will probably find that my perspective as the physician is probably a bit different. For me as a physician the kidney care clinic is great for our patients. It really offloads a lot of the work from us because they are assigned case managers, they have other people who are dealing with the different aspects of their disease; whereas the people I follow in my general nephrology clinic, I'm it. I have no help, I follow everything. I follow their diabetes, I follow all their medications, all their lab work. So from the physician perspective, I mean I recognize the importance of this and I actually think this is a great tool in triaging and deciding who gets to be followed in these like scarce resource clinics, but in terms of workflow, it probably makes our workflow as physicians a bit more heavy only because now we no longer have someone to follow up on some of these things for us. So all the load falls on us which is ok, I think that's the way it should be, but it does mean a bit more work for us.	Nephrologist	Interview	418
	• What happened is we have quite a number of staff off sick, for basically for whatever reason and/or family crisis, we never have full staff. We always have to cover one another, we have no casual, so we have three staff that phone in sick, [the] existing three, remaining three nurses have to double up their workload. So if we only have to case manage our caseload, it's no big deal. It's just when we have to cover others, yeah, like we just don't have enough staff.	Nurse	Interview	424
	• I don't think implementing the KFRE has dramatically impacted [nurses] workload. It has dropped their numbers substantially, yes. But, most of the patients they have discharged from their caseload, I would say probably 80% of the patients were the ones that didn't call them anyway and they were kind of the nice ones, you know you look at the bloodwork, it was fairly stable. You really didn't have to do anything, so this might sound kind of awful, but it's the really acutely ill patients that have been left on the caseload, so you know they had 160 [patients] and now they have a 120 or 130 but they are the really sick 120-130 that you're managing 80% of the time anyway and you know the numbers look great, but it doesn't, it's not indicative of the workload...the workload is still high	Nurse	Interview	420
	• I won't say it's reduced my workload. Like I haven't noticed a real change in my actually day-to-day workload. It's still just as busy. I think I'm not busy with those, I wasn't too busy with those patients anyway because they were such low risk, they weren't calling often. They weren't needing constant interventions, so they weren't time consuming. Certainly not seeing them in clinic, I guess saves a little time, but we were maybe only seeing them every s months because they were so low risk. So, the ones that I have discharged I haven't seen back yet.	Nurse	Interview	423
	• Interviewer: Has your workload changed since April last year as a result of the KFRE implementation?<br>Nurse: no, it didn't change much…actually, in some cases I will say, I actually have less workloac	Nurse	Interview	421
	• My caseload number, [was] 168 [and went] down to, after we transitioned all those people back to geneph, down to about 132 and gradually came down to about 120-some nowadays, 128 I would say. So it's a good number. That will help us to concentrate our effort...we can spend more time with them rather than trying to squeeze everybody in, yeah so it definitely helped with our time	Nurse	Interview	424

Appendix 4 Post KFRE Implementation Exemplary Quotations

Category	Healthcare Providers	Role	Mode	Pt ID
**Job satisfaction**	• I don't think it's as big of a deal, I've heard some people say [it has increased their workload], for me it didn't really change a lot. I think from practice I generally keep people in my general nephrology until they are more severe anyways. I don't send people early. So, I haven't had that many people that needed to be moved away from the [kidney] clinic and so there's been some, but I feel like those were appropriate in general in that it actually made sense because those people weren't really, not a whole lot was happening with them in terms of the kidney disease. So, it actually made sense, so for me, yes it probably did add a little bit of workload just moving the charts and explaining the process to the patients, but in the big picture it probably made things easier because I noticed that whenever I do send patients and the wait times for, and it's perceived and I don't know if there is data on this, but it's perceived that from my perspective getting patients into the kidney clinic seems a little bit faster than it used to be. So it probably made things easier in th big picture, you know the trade off was worth it.	Nephrologist	Interview	407
	• I would say, I actually haven't [noticed a change in workload] and I think the reason is because we've kind of, with the KFRE we've kind of gotten rid of, like I said, the patients that don't really need us as much and maybe weren't even accessing us as much because they did have higher GFRs and didn't have as many concerns. So I'm finding actually the workload hasn't changed much. It's probably the patients that we are keeping are more kind of high needs and do phone us more and do contact us more...I think they just demand more of our attention and our time, so I haven't really noticed a huge difference in our workload.	Nurse	Interview	413
	• The acquity of patients is greater, more challenging	Nurse	Survey	24

Appendix 4 Post KFRE Implementation Exemplary Quotations

Category	Patients & Family Members	Role	Pt ID
Targeted care	• Patient: We have been discussing the issue for six months or once a year for awhile and I assumed when they gave me the letter saying that I was now below the 5% threshold for likely dialysis in the next two years and would not be using the clinic facilities completely but simply meeting with [nephrologist] that would probably would go to the one year [appointment] and when we talked about it this February we decided that would be frequent enough and he is available if something happens. If nothing happens then he can spend the time looking after those who still have more need than I do. It was included in the letter that said that due to this grading, I would no longer make use of the kidney clinic service, but only the nephrologist.<br>Interviewer: and what did you think of that letter?<br>Patient: I thought it was very appropriate. We need to conserve our resources and use them where they are needed and I think at this point in time, that was something I didn't need, so I know there's always more people, there's more demand than we can meet.	Patient	225
	• There's no need for me to see him [my Nephrologist] every time I go in [for bloodwork] because and if I have a question in-between appointments, I know I can phone, so that works pretty well.	Patient	223
	• I think yearly is fine you know, as long as I'm stable there's no point in me going in every three months, so she can tell me that 'yup, everything is stable'.	Patient	228
	• Patient: I'm getting excellent care as far as I'm concerned. I'm going to see him [my Nephrologist] one more time next year and he's serious that may be the last time.<br>Interivewer: ok, and how do you feel about that?<br>Patient: well I do get a comfort feeling of seeing him but I know he's very busy and there are people that are worse condition, if that's the word to use, than I am. So I accept his plan and he indicated if I did have any problems at all to have my family doctor give me a call, or give him a call, explain what the problems are and then he would see me any time.	Patient	231
Patient reassurace and reduced stress	• I mean there is a 4% chance that you would have kidney failure, but on the other hand, 96% that you don't have it, that's kind of reassuring.	Patient	221
	• Having that equation and knowing that the chances of me going into kidney failure in the next 10 years is extremely low was quite reassuring.	Patient	230
	• I miss not talking to them [the nurses], but I'm glad I don't have to do it because it's difficult for me to get there. I'm way out in the south east and the Sheldon Chumir centre is a long way to go to make arrangements for rides and so on like that. I'm not unhappy about not going.	Patient	226
Access to resources outside of the CKD clinic	• Patient: When I do my bloodwork in between [nephrology appointments] I get it from my family doctor rather than from the clinic<br>Interviewer: ok and how has that been going?<br>Patient: it's good because I just have to explain that I've had lab work done in the last week or whatever, he [my family doctor] can look it up on the system now...<br>Interviewer: that's great and are you comfortable talking about your CKD with your family doctor?<br>Patient: yup, no problem at all, he's well aware of it and has kept up even when I was doing the [multidisciplinary] clinic. If there were any questions that I had in-between I was able to get him [my family doctor] to look them up and deal with them.	Patient	225
	• I'm in a residence where we have 24-hour care...we have a nurse practitioner that comes in several times a week...and if we need to see her we are quite free to do that.	Patient	226
	• Interviewer: and do you see your family doctor regarding your CKD?<br>Patient: no he's a family doctor and he really doesn't have a clue. I know that sounds terrible...with the kidney failure you know, he is following [me] because he gets records sent of my bloodwork and everything, so he does know what's going on, but yeah I just need a better family doctor.	Patient	228
	• Interviewer: Do you feel like you have a good relationship with your GP that he or she is able to help you with your concerns relating to kidney disease?<br>Patient: oh definitely, I have a very good relationship with her.	Patient	230

Appendix 4 Post KFRE Implementation Exemplary Quotations

Category	Patients & Family Members	Role	Pt ID
**Access to resources outside of the CKD clinic**	• Patient: I'm going to see him [my Nephrologsit] one more time next year.<br>Interviewer: ok and if that does happen in about a years time, do you feel comfortable that your family doctor will be able to manage your kidney disease?<br>Patient: yes.	Patient	231
	• Interviewer: In terms of accessing any allied health providers, dieticians, pharmacists, social workers, how has that been going?<br>Patient: I don't really have to access [allied health] other than going to my pharmacist...as far as dietitians when I do see one they are pretty helpful, but...everything is pretty stable, cause now I'm up to yearly visits with her [my nephrologist] and nothing has really changed.	Patient	228
**Self-efficacy**	• Interviewer: that's great that you keep track of everything and you have a log. That must be very helpful when you have a visit to be able to reflect.<br>Family Member: yeah I have a grid for each of us that contains the doctors, the surgeries, the health problems, the prescriptions and dates included and it's a grid on two pages back and fourth and it's just wonderful.	Family Member	229
	• We started back a couple of years ago...we have worked in the meantime and followed instructions very closely and it has improved substantially, hasn't it? ...so just being careful and you know keeping his weight, watching the fluid in the legs…	Family Member	229
	• I do a pretty good job of keeping track of what I should eat and should not eat and she has given me a lot of specific information that I follow and it seems to reflect in my levels because they always tell me I'm maintaining my rate, you know, it's not really a high rate of kidney function but it stays the same. So this charting of it helps me know what to eat and what not to eat.	Patient	223
	• Patient: in recent times I haven't had any recourse because I have all the literature and I've been following the diet program, so it's been good for me<br>Interviewer: oh you've been following a diet program?<br>Patient: in a sense of watching, I'm on a medication that is potassium sparing and so potassium and salt intake are my two major issues and I avoid most preserved foods and canned goods and do mostly fresh vegetables and meat products, so that has helped a lot.	Patient	225
	• I used to see [the Dietitian] but there's no concerns and I more or less said that I really don't need to see them...I'm eating differently and I'm able to function now, yeah I don't really see them anymore.	Patient	228
	• I would use [a patient portal to access lab results], absolutely, that way I don't have to wait and see her [my nephrologist] to see what my GFR, what my potassium's at or whatever or my sodium or my acid...I think would be a big help because then I can do things before I see her...then you don't have to wait for the doctor's appointment for her to say, well maybe you should try this, you know you can get on it right away.	Patient	228
	• Patient: Going to a GP just to get lab results seems a little bit excessive...[A patient portal to access lab results] would be marvellous. I know my brother used to live in BC and he said that they each had an ID number and if they wanted to look up a result they could and now I have to go to my GP to get the results.<br>Interviewer: Would you be comfortable going online and looking at your labs?<br>Patient: oh definitely	Patient	230
**Transition process**	• Interviewer: and so if you had trouble with your kidney's and you had a question now, who would you call?<br>Patient: I guess first of all I would call my local doctor...the kidney clinic and the heart clinic both said [you're] fine, you've graduated, you're discharged. But, they said if there was a concern or a problem that I was to phone them immediately which is nice to know, but that hasn't occurred.	Patient	220
	• The nurse that looked after me, she phoned every once in awhile, and so she checked my tests and she would phone me if there was a problem, or she would just phone me to tell me that everything was all right. That I appreciated...I think it would be better if I did hear [from the Nurse].	Patient	221

Appendix 4 Post KFRE Implementation Exemplary Quotations

Category	Patients & Family Members	Role	Pt ID
**Transition process**	• Patient: I did rely on my nurse manager quite a bit. She was my go-to person, and I knew when I phoned her in the morning and left a message, she would get back to me that afternoon as soon as she was free. So, I don't have that anymore, but [my Nephrologist] has made sure I have a phone number to phone if I need something, but it's not as direct as dealing with [the nurse case manager].<br>Interviewer: and now that you have this number, have you used it?<br>Patient: no, I haven't had to.	Patient	223
	• Interviewer: In the past, did you call your nurse case manager quite a bit?<br>Patient: nope, I'd only call her if I wanted to know the results of the bloodwork.<br>Interviewer: ok and so in the last year [since the transition to general nephrology] have you called anybody else if you were wanting to know about results or if you have any questions?<br>Patient: no.	Patient	226
	• Interviewer: How do you feel about not having access to the nurse manager now?<br>Patient: I don't want to be negative, but sometimes I feel like I've been thrown to the wolves…actually it came as a surprise to me, I wasn't really expecting it, but I guess a little more information or explanation at that time might have been a little bit helpful, I was just told I was stable and being stable for a few years so I was just getting moved down an that was it.	Patient	230
**Job satisfaction**	• n/a		
**Anticipated concerns**	• n/a		
