# Supplementary material for: Patient and provider experience and perspectives of a risk-based approach to multidisciplinary chronic kidney disease care: a mixed methods study
Source: BMC Nephrol. 2019 Mar 29;20:110. doi: 10.1186/s12882-019-1269-2 (PMC6440153; doi:10.1186/s12882-019-1269-2)
Supplement: Supplementary file 5 — Pre Post Categories Summary: A summary of the pre- and post-implementation content categories reflecting our directed content analysis approach to the data. (PDF 72 kb) [file 12882_2019_1269_MOESM5_ESM.pdf]

Appendix 5. Post-Implementation KFRE Qualitative Analysis – Pre/Post Content Category Summary

Pre-implementation category	Post-implementation category	Description
Focused Resources<br>Sustainability<br>Targeted Care	**Targeted Care**	No change from pre-implementation study. Includes references to personalized medicine, targeting care to patients based on individualized needs, ensuring patients are seen in most appropriate setting, and sustainability of care model.
Reduce Patient Stress	**Patient Reassurance and Reduced Stress**	The KFRE model may decrease burden on lower-risk patient's emotional/mental health and physical well-being because they will be exposed to fewer unnecessary laboratory tests, follow-up visits, and will be aware that their disease trajectory will not likely require renal replacement therapy. For the post-implementation study, the definition is expanded to include references to reassurance gained from knowing KFR value.
Build other HCP capacity<br>Education & Capacity outside of CKD clinic	**Access to Resources Outside of the CKD Clinic**	Includes references to opportunities to build capacity for other providers to care for CKD patients in the community. The idea that CKD clinics are operating beyond their scope, fulfilling roles that could be more appropriately performed by other providers, such as primary care, internal medicine, or through primary care networks (PCNs). Includes examples of where multidisciplinary resources already exist outside of the CKD clinic (PCN teams, for example) and patient/provider awareness and comfort in accessing those resources.
Encourage Patient Self-Management	**Self-efficacy**	In the pre-implementation study, referred to lack of self-management strategies, self-advocacy, poor health literacy, and the idea that the KFRE care model will increase self-management because lower risk patients won't be able to rely on the case manager. In the post-implementation study, this includes patient/provider comments or examples of patient self-management following KFRE implementation. Also, expanded to include access to laboratory values to facilitate self-management.
Access to NCM<br>Access to Prev. Ed<br>Disease Monitoring<br>Distrust of KFRE	**Anticipated Concerns**	Positive/negative/neutral comments relating to lost access to nurse case manager (lower risk patients) and preventive education offered at the CKD multidisciplinary clinic. Also includes perceived concerns about the KFRE predictive ability and long-term outcomes for lower-risk patients (slowing progression, borderline patients/fluctuating KFR, Patient complexity and co-morbidities).
Communication<br>Change Process	**Transition Process for Low Risk Patients**	Communication issues related to the transition/change process (may relate to internal/external communication with patients/other providers, changes to blood work requisitions, location of paper charts etc. Also includes patient or provider reluctance to discharge lower risk patients to general nephrology or strategies use to facilitate transition.
n/a	**Job Satisfaction**	References to job satisfaction and workload changes resulting from KFRE implementation.
